# Validation of adult asthma case definitions for primary care sentinel surveillance

**DOI:** 10.1186/s13223-023-00854-8

**Published:** 2023-11-13

**Authors:** Max Moloney, Alison Morra, Rachael Morkem, John Queenan, Samir Gupta, Teresa To, Geneviève Digby, David Barber, M. Diane Lougheed

**Affiliations:** 1 Asthma Research Unit, Kingston General Hospital, Kingston Health Sciences Centre at Queen’s University, 72 Stuart Street, Kingston, ON K7L 2V7 Canada; 2 Division of Respirology, Department of Medicine, Queen’s University, Kingston, ON Canada; 3 Department of Family Medicine, Queen’s University, Kingston, ON Canada; 4 Canadian Primary Care Sentinel Surveillance Network (Eastern Ontario Network), Kingston, ON Canada; 5 Division of Respirology, Department of Medicine, St. Michael’s Hospital, Toronto, ON Canada; 6 Department of Medicine, University of Toronto, Toronto, ON Canada; 7 Child Health Evaluative Science, The Hospital for Sick Children, Toronto, ON Canada; 8 Dalla Lana School of Public Health, University of Toronto, Toronto, ON Canada

**Keywords:** Asthma, Electronic medical records, Quality improvement, Knowledge translation

## Abstract

**Background:**

Most asthma diagnoses and patient care take place in primary care settings. Electronic medical records (EMRs) offer an opportunity to utilize technology to improve asthma diagnosis and care. The purpose of this study was to create and validate separate case definitions for suspected and confirmed asthma in primary care EMRs, to enable surveillance, benchmarking, and quality improvement in primary care settings. The objective of this study was to develop a case definition for suspected and confirmed asthma for use in a primary care sentinel surveillance system.

**Methods:**

A single chart abstractor conducted a manual audit of 776 randomly selected patient charts from an academic primary care practice EMR in Kingston, Ontario. Following the single chart abstractor classification, a consensus on chart classification as “not asthma”, “suspected asthma”, or “confirmed asthma” was achieved between the abstractor, a family physician, and a respirologist using Canadian Thoracic Society (CTS) criteria. Case definition algorithms based on billing codes, clinical data elements and medications were applied to the site’s Canadian Primary Care Sentinel Surveillance Network (CPCSSN) data for the same charts and compared to abstractor classifications to determine each algorithm’s measurement properties.

**Results:**

The prevalence of suspected and confirmed asthma were 7.3% (n = 54) and 2.4% (n = 18), respectively. None of the proposed case definitions could differentiate between suspected and confirmed asthma. One algorithm consisting of billing, clinical, and medication elements had the highest Youden’s Index for either suspected or confirmed asthma. The algorithm had a sensitivity of 81%, a specificity of 96%, positive predictive value of 71%, negative predictive value of 98%, and a Youden’s Index of 0.77 for combined suspected or confirmed asthma cases.

**Conclusion:**

An EMR case definition for suspected or confirmed adult asthma has been validated for use in CPCSSN. Implementation of this case definition will enable the development of a surveillance electronic tool (eTool) for adult asthma that can foster quality improvement.

**Supplementary Information:**

The online version contains supplementary material available at 10.1186/s13223-023-00854-8.

## Background

Asthma is diagnosed based on a combination of patient history, physical examination, and objective tests. There are over 3 million Canadians diagnosed with asthma and the prevalence of asthma in Canada is approximately 8.5% [1]. The Canadian Thoracic Society (CTS) defines asthma as the combination of a compatible clinical history of asthma and reversible airflow obstruction or airway hyperresponsiveness on lung function tests, or alternatively, a specialist diagnosis of asthma [2, 3]. Accordingly, although confirmed asthma requires objective testing, a “suspected asthma” case is defined as a compatible clinical history without objective measures of lung function consistent with asthma or specialist diagnosis [2, 3]. Most asthma diagnoses and care occur in primary care settings [4]. Despite available guidelines, less than half of patients diagnosed with asthma have undergone appropriate pulmonary function testing to confirm their diagnosis [5]. As such, the majority of real-world diagnoses in primary care are more accurately classified as cases of suspected asthma. This failure to use objective testing has led to a high degree of misdiagnosis of asthma [6].

Electronic medical records (EMRs) offer an opportunity to utilize technology to improve the process of asthma diagnosis. Potential benefits of using electronic tools (eTools) within EMRs include improved quality of care, outcome monitoring, and performance measurement [7, 8]. The Canadian Primary Care Sentinel Surveillance Network (CPCSSN) is the first and only pan-Canadian chronic disease surveillance system based on primary care EMR data [9]. CPCSSN has validated case definitions for 13 chronic conditions, including COPD [10]. There have also been efforts to create case definitions for adult asthma using EMR data directly by Xi et al. [11], and using EMR data extracted into the CPCSSN database by Cave et al. [12]. To date, there remains no standardized case definition for diagnosis of asthma in primary care that can be applied to EMRs and databases across Canada. As primary care EMR data are increasingly being used for disease surveillance, validated case definitions are required [13, 14]. A recent literature review on asthma case definitions identified the need to create a case definition that differentiates between suspected and confirmed asthma in primary care EMRs [15]. The purpose of this study was to create and validate separate case definitions for suspected and confirmed asthma in adults in primary care EMRs, and to determine the ability to distinguish between suspected and confirmed asthma using primary care EMR data.

## Methods

### Study design

A retrospective chart analysis was conducted at the Queen’s Family Health Team (QFHT) in Kingston, Ontario. The QFHT uses the open-source OSCAR EMR developed by McMaster University that is used across Canada, in the care of over 1 million patients [16]. CPCSSN collects de-identified patient data from source EMRs, including but not limited to demographics, visit dates, reason for encounter, medical conditions, billing history, procedure history, prescribed medications, laboratory results, and patient referrals. This study used coded and processed QFHT data in CPCSSN, stored at the Centre for Advanced Computing at Queen’s University. The random sample in this study was derived from a list of patient charts from CPCSSN data holdings generated by a computer algorithm. Patients that elected to opt-out of research from CPCSSN were removed from the list of patient charts for review.

QFHT patients were notified of the study and given the option to withdraw from the study. The study was approved by the Health Sciences and Affiliated Teaching Hospitals Research Ethics Board (HSERB) at Queen’s University. All data were recorded as non-identifiable information.

### Chart abstraction and data collection

The criteria for generation of the patient list were age ≥ 18 years and currently registered at QFHT. Charts were excluded if there was no recorded visit to a physician in the previous 5 years or if a chart was marked as inactive by QFHT. A single chart abstractor conducted a manual audit of the 776 randomly selected patient charts for 776 patients. The sample of 776 patient charts was determined based on a power calculation assuming 8% prevalence of asthma in adults [17, 18], along with a projected case definition algorithm with 80% sensitivity, 10% precision and 95% CIs. Given these parameters, the minimum sample size was determined to be 776. The abstractor collected 96 data points selected a priori by consensus of the lead investigators relevant to asthma diagnosis and management, and classified each chart as “not asthma”, “suspected asthma”, or “confirmed asthma”. The categories of data collected included patient demographics, prescribed medications, asthma symptom history, comorbidities, smoking status, documentation of asthma diagnosis, asthma exacerbation history, pulmonary function tests, and referral notes. Data collected included a lookback time frame of the entire patient’s history, with the exception of medication data, which was separated into expired (> 2 years) and current (≤ 2 years). The abstractor used a chart abstraction manual to ensure data entry accuracy and consistency. All data were collected using an online abstraction form created on Qualtrics™ software.

### Asthma classification definition

The definition of “confirmed asthma” was based on the CTS guideline for asthma diagnosis [2, 3]. Confirmed asthma was defined as a compatible clinical history plus pulmonary function tests (PFTs) confirming asthma, and/or a specialist diagnosis. “Suspected asthma” was defined as a compatible clinical history without PFTs consistent with asthma or a specialist diagnosis. All other patient charts were classified as “not asthma.” Following the single chart abstractor classification, each chart classified as suspected or confirmed asthma and 12 charts classified as not asthma which the abstractor thought required additional input for classification, were reviewed by a QFHT family physician and a respirologist to achieve consensus on the final classification. All chart classification reviews were completed on the original OSCAR EMR charts at QFHT.

### Case definition development

Case definitions were developed and tested on the CPCSSN data holdings for the same charts reviewed and classified by the abstractor and expert physicians in the OSCAR EMR at QFHT. Case definitions tested in this study were developed by members of the study team. They included 3 case definitions for adult asthma previously published in the literature, in addition to new case definitions designed by the research team [11, 12]. The proposed case definitions were developed based on all data fields possibly relevant for diagnosis in CPCSSN, including billing information, health condition, encounter diagnosis, and medication data. In total, 21 case definitions were developed and tested through an iterative process. Case definition criteria within the search fields used a combination of: text strings (in encounter diagnosis or health condition fields); International Classification of Disease, Ninth Revision (ICD-9) codes (used by QFHT) in billing diagnosis, encounter diagnosis, or health condition fields); and medication prescription information of the CPCSSN data. The complete list of case definitions and the specific search criteria tested are available in Table 1.

**Table 1:** Sample Characteristics (n = 743)

Characteristic	n	Mean (SD)	Median (Range)
Age, years	743	50.3 (8.7)	48.7 (18–93)
	Subcategory		n (%)
Sex	Male		327 (44)
	Female		416 (56)
Smoking History	Never		345 (46)
	Past		206 (28)
	Current		192 (26)
Comorbidity	Allergic rhinitis		76 (10)
	ACOS		10 (1)
	Bronchiectasis		8 (1)
	Chronic bronchitis		17 (2)
	Congestive heart failure		25 (3)
	COPD		47 (6)
	Cor pulmonale		0 (0)
	Emphysema		11 (2)
	GERD		235 (32)
	Sleep apnea		87 (12)
Medication	Expired n (%)	Current n (%)	Total (Expired + Current) n (%)
SABA	98 (13)	96 (13)	194 (26)
SAMA	3 (0)	3 (0)	6 (1)
SAMA/SABA	0 (0)	0 (0)	0 (0)
ICS	77 (10)	45 (6)	122 (16)
LABA	3 (0)	5 (1)	8 (1)
LAMA	4 (1)	9 (1)	13 (2)
ICS/LABA	10 (1)	27 (4)	37 (5)
LAMA/LABA	0 (0)	3 (0)	3 (0)
ICS/LABA/LAMA	0 (0)	1 (0)	1 (0)
LTRA	1 (0)	0 (0)	1 (0)
Biologics	1 (0)	8 (1)	9 (1)
Systemic steroids	50 (7)	39 (5)	89 (12)
Other	50 (7)	673 (91)	723 (97)
Location		n (%)	
Asthma in cumulative patient profile		73 (10)	
Asthma in disease registry		38 (5)	
Asthma referral notes		39 (5)	
Asthma in free text		252 (34)	
Asthma elsewhere in patient chart		34 (5)	
Other asthma documentation data		n (%)	
Asthma synonyms in patient chart		88 (12)	
Work-related asthma in patient chart		12 (2)	
Family history of asthma in patient chart		25 (3)	
Childhood diagnosis of asthma in patient chart		49 (7)	
Use of objective measures of lung function		n (%)	
Spirometry performed		114 (15)	
Bronchodilator challenge test performed		86 (11)	
Methacholine challenge test performed		15 (2)	
Exercise challenge test performed		4 (1)	
Method of confirmation of asthma diagnosis		n (%)	
Specialist diagnosis		3 (0)	
Spirometry		13 (1)	
Methacholine challenge test		3 (0)	
Exercise challenge test		0 (0)	

*ACOS* Asthma-COPD Overlap Syndrome, *COPD* Chronic Obstructive Pulmonary Disease, *GERD* Gastroesophageal Reflux Disease

*ICS* Inhaled corticosteroid, *LABA* Long-acting β-agonist, *LAMA* Long-acting muscarinic antagonist, *LTRA* Leukotriene receptor antagonist, *SABA* Short-acting β-agonist, *SAMA* Short-acting muscarinic antagonist

### Statistical analysis

The results of the proposed case definitions were compared to the confirmed asthma classification definition (reference standard). For each proposed case definition, sensitivity (SN), specificity (SP), positive predictive value (PPV), negative predictive value (NPV), and Youden’s Index (YI) [(sensitivity + specificity) – 1] were calculated in the (i) confirmed asthma subset, (ii) suspected asthma subset, and (iii) combined confirmed or suspected asthma subset. An ROC curve was plotted for combined confirmed or suspected asthma. Additionally, descriptive statistics were calculated for each of the 96 data points abstracted during the study. Statistical analysis was completed using Microsoft Excel™ and SAS™ software.

## Results

### Sample characteristics

**Fig. 1:**
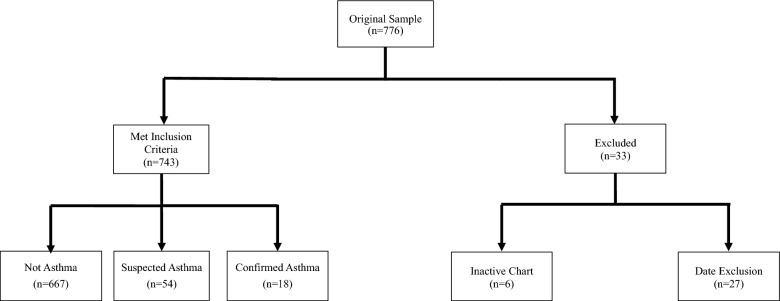
Sample derivation

A total of 743 patient charts were included in the study's final analysis. Thirty-three charts were excluded (Fig. 1). The date cut off for chart inclusion was an entry into the EMR within the last two years.

The characteristics of the sample are detailed in Table 1. The estimated prevalence of suspected or confirmed asthma based on the 743 charts that met inclusion criteria was 9.7% (n = 72). Of the 72 charts determined to have suspected or confirmed asthma, 54 (7.4%) were classified as suspected and 18 (2.3%) were classified as confirmed.

In the study sample, 416 of 743 (56.0%) patients’ charts reviewed were female and 327 charts (44.0%) reviewed were male. The mean (± SD) age of patients reviewed was 50.3 (± 8.7). Additional patient characteristics are outlined in Additional file 1: Table S2. In assessing rates of objective measures to confirm asthma diagnosis, spirometry was completed in 114 (15.3%) of charts reviewed. Completion of spirometry with bronchodilator testing was documented in 86 charts (11.6%). Documentation of a methacholine challenge test was completed in 15 charts (2.0%)., and evidence of a specialist diagnosis was present in 22 charts (3.0%).

### Case definition results

**Fig. 2:**
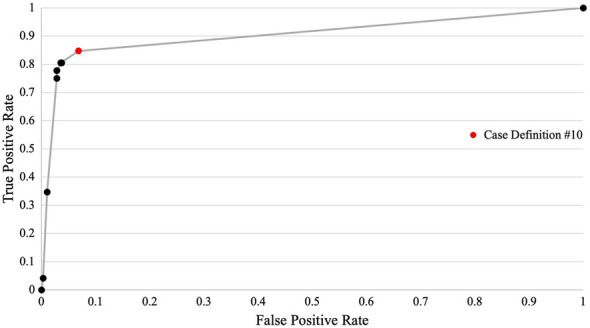
ROC curve of proposed case definitions for suspected and confirmed asthma

The case definition algorithm determined to have the highest Youden’s Index for the combination of suspected or confirmed asthma was Case Definition 10, which used a combination of text strings and ICD-9 codes from the billing, encounter diagnosis, and health condition within CPCSSN (Table 2 and Fig. 2). This definition had a SN of 81%, a SP of 96%, PPV of 71%, NPV of 98%, and a Youden’s Index of 0.77. For suspected asthma, the case definition had a SN of 76%, a SP of 94%, PPV of 50%, NPV of 98%, and a Youden’s Index of 0.70. For confirmed asthma, the case definition had a SN of 94%, a SP of 91%, PPV of 21%, NPV of 99%, and a Youden’s Index of 0.85. None of the case definitions assessed in this study met the minimum standard (sensitivity and specificity > 70%) to differentiate between suspected and confirmed asthma. Complete results are available in Additional file 1: Table S3–S5.

**Table 2:** Case definition criteria and results

Case Definition	Inclusion Criteria (Any One Of)					Exclusion criteria	SN	SP	PPV	NPV	YI	
	ICD-9 billing	Encounter diagnosis		Health condition	Medications							
1*	1 of: 493						0.35	0.99	0.78	0.93	0.34	
2		1 of: “asthma” OR 1 of: 493 (asthma)					0.75	0.97	0.74	0.97	0.72	
3*				1 of: “asthma” OR 1 of: 493 (asthma)			0.04	1.00	0.60	0.91	0.04	
4	1 of: 493	1 of: “asthma” OR 1 of: 493 (asthma)					0.81	0.96	0.70	0.98	0.77	
5	1 of: 493	1 of: “asthma” OR 1 of: 493 (asthma)				Billing Any of: 491 (chronic bronchitis), 492 (emphysema), 496 (COPD)	0.81	0.96	0.71	0.98	0.77	
6	1 of: 493	1 of: “asthma” OR 1 of: 493 (asthma)				Encounter Diagnosis Any of: “asthma*query” “query*asthma” “asthma*?” “?*asthma”	0.81	0.96	0.70	0.98	0.77	
7	1 of: 493	1 of: “asthma” OR 1 of: 493 (asthma)				Billing Any of: 491 (chronic bronchitis), 492 (emphysema), 496 (COPD) AND/OR Encounter Diagnosis Any of: “asthma*query” “query*asthma” “asthma*?” “?*asthma”	0.81	0.96	0.71	0.98	0.77	
8	1 of: 493	1 of: “asthma” OR 1 of: 493 (asthma)		1 of: “asthma” OR 1 of: 493 (asthma)			0.81	0.96	0.70	0.98	0.77	
9	1 of: 493	1 of: “asthma” OR 1 of: 493 (asthma)		1 of: “asthma” OR 1 of: 493 (asthma)		Billing Any of: 491 (chronic bronchitis), 492 (emphysema), 496 (COPD)	0.81	0.96	0.71	0.98	0.77	
10	1 of: 493	1 of: “asthma” OR 1 of: 493 (asthma)		1 of: “asthma” OR 1 of: 493 (asthma)		Encounter Diagnosis Any of: “asthma*query” “query*asthma” “asthma*?” “?*asthma”	0.81	0.96	0.70	0.98	0.77	
11	1 of: 493	1 of: “asthma” OR 1 of: 493 (asthma)		1 of: “asthma” OR 1 of: 493 (asthma)		Billing Any of: 491 (chronic bronchitis), 492 (emphysema), 496 (COPD) AND/OR Encounter Diagnosis Any of: “asthma*query” “query*asthma” “asthma*?” “?*asthma”	0.81	0.96	0.71	0.98	0.77	
12	2 of: 493	2 of: “asthma” OR 2 of: 493 (asthma)		1 of: “asthma” OR 1 of: 493 (asthma)			0.78	0.97	0.75	0.98	0.75	
13†	2 of: 493	2 of: “asthma” OR 2 of: 493 (asthma)		1 of: “asthma” OR 1 of: 493 (asthma)		Encounter Diagnosis/Health Condition “asthma*query” “query*asthma” “asthma*?” “?*asthma”	0.78	0.97	0.75	0.98	0.75	
14	1 of: 493	1 of: “asthma” OR 1 of: 493 (asthma)		1 of: “asthma” OR 1 of: 493 (asthma)	Any medication from asthma medication list	Medications LAMA (monotherapy) LABA (monotherapy) LAMA/LABA (monotherapy)	0.85	0.93	0.57	0.98	0.78	
Medication Case Definition	Inclusion Criteria (Any One Of)					Exclusion Criteria	SN	SP	PPV	NPV		YI
	#1		#2									
M-1	SABA		Budesonide/formoterol			Any other respiratory medication on medication list	0.35	0.96	0.47	0.93		0.31
M-2	SABA + ICS		SABA + LTRA			Any other respiratory medication on medication list	0.10	0.98	0.39	0.91		0.08
M-3	ICS					Any other respiratory medication on medication list	0.08	0.98	0.32	0.91		0.06
M-4	SABA + ICS/LABA					Any other respiratory medication on medication list	0.18	0.99	0.93	0.92		0.17
M-5	SABA + (ICS/LABA/LAMA OR ICS/LABA + LAMA OR ICS/LABA + LTRA OR ICS/LABA/LAMA + LTRA)		ICS/LABA/LAMA OR ICS/LABA + LAMA OR ICS/LABA + LTRA OR ICS/LABA/LAMA + LTRA			Any other respiratory medication on medication list	0.08	0.99	0.67	0.91		0.07
M-6	Biologics					Any other respiratory medication on medication list	0	0	0	0		-
M-7	Any medication from asthma medications list					LAMA (monotherapy) LABA (monotherapy) LAMA/LABA (monotherapy)	0.76	0.9	0.45	0.97		0.66

*Adapted from Xi et al. (2015)

†Adapted from Cave et al. (2020)

*LABA* Long-acting β-agonist, *LAMA* Long-acting muscarinic antagonist

*TP* True Positive, *FP* False Positive, *FN* False Negative, *TN* True Negative, *SN* Sensitivity, *SP* Specificity, *PPV* Positive Predictive Value, *NPV* Negative Predictive Value, *YI* Youden’s Index

*ICS* Inhaled corticosteroid, *LABA* Long-acting β-agonist, *LAMA* Long-acting muscarinic antagonist, *LTRA* Leukotriene receptor antagonist, *SABA* Short-acting β-agonist, *SAMA* Short-acting muscarinic antagonist

*TP* True Positive, *FP* False Positive, *FN* False Negative, *TN* True Negative, *SN* Sensitivity, *SP* Specificity, *PPV* Positive Predictive Value, *NPV* Negative Predictive Value, *YI* Youden’s Index

## Discussion

We validated a case definition for combined suspected or confirmed asthma in primary care. This study's proposed case definitions had similar results for both suspected and confirmed asthma. Case definitions could not discriminate between suspected and confirmed asthma because the use of objective measures to confirm asthma diagnosis was either not completed or not documented. Our findings of a combined prevalence of suspected and confirmed asthma of 9,7% is comparable to current national statistics [18]. However, 75% of the cases in our study were suspected not confirmed. This highlights the importance of confirming and documenting the status of asthma diagnoses in EMRs. National statistics based on population surveys that rely on self-report of physician diagnosis or billing data may also be subject to considerable misclassification. Until EMR data elements are adopted that allow for the distinction between suspected and confirmed asthma [19], one case definition that can be used for combined suspected or confirmed asthma is recommended.

Our proposed case definitions had similar operating characteristics to those reported previously. However, in replicating case definition algorithms from both Xi et al. [11] and Cave et al. [12] (Table 1), we found different results across all metrics calculated. For example, for Case Definition 1, Xi et al. (2015) report a SN of 78% and SP of 89%, compared to a SN of 35% and SP of 99% in our study. For Case Definition 3, Xi (2015) reported a SN of 7% and SP of 99%, compared to a SN of 4% and SP of 100% in our study [11]. Case Definitions 1 and 3 were attempts to replicate their algorithms and were considered approximated because the original case definition algorithms used information directly from the source EMR in OSCAR. For Cave et al., the metrics were similar, with a reported a SN of 83%, SP of 99%, PPV of 74%, NPV of 99%, and a Youden’s Index of 0.82, compared to a SN of 78%, SP of 97%, PPV of 75%, NPV of 98%, and YI of 0.73 in our study. [12]

This variability can likely be attributed to the variation in the data sources used for case definition analysis and the variation in charting behaviour between clinical sites. Xi et al. (2015) created a cohort with a high proportion of patients with asthma and COPD for analysis. In contrast, we used a population-based sample, thus having a lower asthma prevalence, reducing SP and PPV while improving SN and NPV. In Cave et al.’s (2020) study, the authors used data from the Southern Alberta Primary Care Research Network of CPCSSN (SAPCReN-CPCSSN) to classify cases of asthma. In this study, reviewers used the source EMR for classification, allowing for a complete review of the patient’s entire medical history.

The results of this study highlight the importance of having discrete data elements for asthma diagnostic tests in EMRs, particularly given that there were no searchable data elements that enabled us to differentiate between suspected and confirmed asthma. In addition, in EMRs, there is no requirement for confirming asthma diagnosis through objective measures such as spirometry or a methacholine challenge test. EMRs should incorporate data elements such as those proposed by the Pan-Canadian Respiratory Standards Initiative for Electronic Health Records (PRESTINE) so that providers are able to document whether asthma is suspected or confirmed, and if confirmed by what method [7, 20]. Data elements that capture if asthma has been confirmed would enable case definition search strategies to differentiate between suspected and confirmed asthma [19]. By adopting these data elements, knowledge translation eTools could provide decision support to healthcare providers on cases of suspected asthma that require objective testing, while simultaneously improving asthma surveillance by ensuring cases of asthma are confirmed asthma [21].

In our study, although we included every medication combination presented in the CTS guidelines for asthma management [3] (Case Definitions M1-M7), medication data did not improve the operating characteristics of detection algorithms (Table 1). The proposed case definitions that included medication data had a wide sensitivity range, from 0 to 76%. This result differs from previous literature on asthma case definitions, which discuss adding medications as an effective way to improve case definitions [22]. We believe that this may be because many medications are now being used for both asthma and COPD, and as could contribute to misdiagnosis of asthma and COPD if used as part of EMR algorithms. Additionally, this finding suggests that researchers creating asthma case definitions must be very specific in their inclusion or exclusion of medications in case definitions.

The findings of our study fit well within the existing literature on the validation of asthma diagnoses using EMRs. A recent study from Howell et al. [23] developed a case definition algorithm for asthma using EMR data from a pulmonary specialty clinic. This study’s best-case definition had a SN of 94% and a SP of 85%. These results are slightly higher than the results of our study. In this case, the slightly higher SN and SP can be attributed to using a specialty clinic, which would be more likely to have confirmed cases of asthma, improving specificity, and a higher relative proportion of patients with asthma, improving sensitivity. Another systematic review of literature on the validation of asthma diagnoses in electronic health records by Nissen et al. described 13 studies on the subject [22]. The authors found that most studies were able to demonstrate a high positive predictive value (PPV > 80%), with a high degree of variation based on methodology used. Our study builds upon the systematic review by using a national database that can utilize the case definition in primary care practices across Canada.

We were able to directly replicate the case definition proposed by Cave et al., given that it also used CPCSSN data holdings. For case definition 13, Cave et al. (2020) reported a SN of 83% (+ 5%), a SP of 99% (+ 2%), PPV of 74% (-1%), NPV of 99% (-1%), and a Youden’s Index of 0.82 (+ 0.09), which are nearly identical to our results. The discrepancy between the findings can be attributed to the data source used for classifying cases of asthma and the data source used for validating the case definition.

The clinical implications of using a combined case definition for asthma in primary care EMRs for suspected and confirmed asthma are important to consider. Until EMR data elements that document whether asthma has been confirmed by objective lung function tests are widely adopted, surveillance data utilizing an asthma EMR case definition that cannot differentiate between suspected and confirmed asthma may over-estimate true asthma prevalence. Separate case definitions would provide more accurate information on disease patterns, prevalence, and performance measurement for quality improvement. Future knowledge translation initiatives should focus on adoption of EMR data elements that would allow separate EMR case definitions for the suspected and confirmed asthma.

### Strengths

Strengths of this study include using the original EMR source data for chart abstraction and classification. By manually reviewing the patient chart, the abstractor and physicians had the entire medical record of a patient available to accurately classify the charts based on all information available. Another strength of this study is the use of CPCSSN data holdings for testing and validating case definitions. CPCSSN data is more granular than health administrative data that has been used for case definitions of asthma in the past. This is due to CPCSSN’s data being derived from primary care medical records which have more specific information than health administrative data. In addition to CPCSSN’s added specificity, CPCSSN remains more broadly applicable than data from a single EMR as it compiles data from multiple EMR platforms [24]. Another strength of utilizing CPCSSN as a database is to improve the generalizability of the study, as CPCSSN can analyze data from all major EMR providers in Canada. This allows the proposed case definition to be applied across the country to various primary care settings and EMR providers. Additional strengths of this study are the use of a single abstractor and experts for classification purposes, which ensured consistency in both data collection and final classification of cases.

### Limitations

Limitations of this study include generalizability and the data source. This exercise was conducted at a single academic clinical site that is a member of CPCSSN. It may be difficult to generalize the findings at this academic primary care practice to community practices, as the case mix may differ, and this particular practice may have unique charting, billing, and data entry patterns. Additionally, this study used information from one EMR, OSCAR. As a result, the case definitions developed in this study may have different results when applied to other EMRs, although the criteria used in the CPCSSN database applies to sites across Canada.

## Conclusion

An EMR case definition for confirmed or suspected adult asthma has been validated against original primary care EMR data for use in primary care, including CPCSSN. Implementation of this case definition will enable surveillance and quality improvement of adult asthma care in primary care sites across Canada. Currently, it is not possible to differentiate between suspected and confirmed asthma in primary care EMRs or CPCSSN datasets. As such, adoption of Pan-Canadian standards for EMR elements and algorithms, as proposed by PRESTINE, that identify suspected but unconfirmed asthma and prompt further investigations, is critical to improving the diagnostic accuracy of primary care surveillance and quality improvement systems. Incorporating these data elements into EMR platforms will enable validation of more specific asthma case definitions, and improve surveillance, and quality improvement opportunities for primary care practices.

### Supplementary Information

**Additional file 1:** **Table S1. **Asthma Medications List. **Table S2. **Additional Sample Characteristics (n=743). **Table S3. **Case Definition Results (Combined Suspected or Confirmed Asthma). **Table S4. **Case Definition Results (Suspected Asthma). **Table S5. **Case Definition Results (Confirmed Asthma).

## Data Availability

The datasets used during the study are available from the corresponding author upon reasonable request.

## Abbreviations

CPCSSN: Canadian Primary Care Sentinel Surveillance Network
CTS: Canadian Thoracic Society
EMR: Electronic medical records
eTool: Electronic tools
HSERB: Health Sciences and Affiliated Teaching Hospitals Research Ethics Board
ICD-9: International Classification of Disease, Ninth Revision
NPV: Negative predictive value
PPV: Positive predictive value
PRESTINE: Pan-Canadian Respiratory Standards Initiative for Electronic Health Records
QFHT: Queen’s University Family Health Team
SN: Sensitivity
SP: Specificity
YI: Youden’s index
