# Supplementary material for: Validation of adult asthma case definitions for primary care sentinel surveillance
Source: Allergy Asthma Clin Immunol. 2023 Nov 13;19:95. doi: 10.1186/s13223-023-00854-8 (PMC10644606; doi:10.1186/s13223-023-00854-8)
Supplement: Supplementary file 1 — Additional file 1: Table S1. Asthma Medications List. Table S2. Additional Sample Characteristics (n=743). Table S3. Case Definition Results (Combined Suspected or Confirmed Asthma). Table S4. Case Definition Results (Suspected Asthma). Table S5. Case Definition Results (Confirmed Asthma). [file 13223_2023_854_MOESM1_ESM.docx]

**Additional file 1**

**Table S1.** Asthma Medications List

| **Drug Class** | **Drug Name** | **Brand Name** | **ATC Code** |
| --- | --- | --- | --- |
| Biologics | Benralizumab | Fasenra® | R03DX10 |
|  | Dupilumab | Dupixent® | N/A |
|  | Mepolizumab | Nucala® | R03DX09 |
|  | Omalizumab | Xolair® | R03DX05 |
|  | Reslizumab | Cinqair® | R03DX08 |
| ICS | Beclomethasone | QVAR® | R03BA01 |
|  | Budesonide | Pulmicort® | R03BA02 |
|  | Ciclesonide | Alvesco® | R03BA08 |
|  | Fluticasone Furoate | Arnuity® | R03BA09 |
|  | Fluticasone Proprionate | Flovent® | R03BA05 |
|  | Mometasone | Asmanex® | R03BA07 |
| ICS/LABA | Budesonide/Formoterol | Symbicort® | RK03AK07 |
|  | Fluticasone/Salmeterol | Advair® | R03AK06 |
|  | Fluticasone/Vilanterol | Breo® | R03AK10 |
|  | Indacaterol Acetate/Mometasone Furoate | Atectura® | R03AK14 |
|  | Mometasone/Formoterol | Zenhale® | R03AK09 |
| ICS/LAMA/LABA | Fluticasone Furoate/Umeclidinium/Vilanterol | Trelegy® | R03AL08 |
|  | Indacaterol Acetate/Glycopyrronium Bromide/Mometasone Furoate | Enerzair® | R03AL12 |
| LABA | Formoterol | Oxeze® | R03AC13 |
|  | Indacaterol | Onbrez® | R03AC18 |
|  | Salmeterol | Serevent® | R03AC12 |
| LABA/LAMA | Aclidinium/Fomoterol | Diskair® | R03AL05 |
|  | Indacaterol/Glycopryronium | Onbrez® | R03AL04 |
|  | Tiotropium/Olodaterol | Inspiolto® | R03AL06 |
|  | Umeclidinium/Vilanterol | Anoro Ellipta® | R03AL03 |
| LAMA | Aclidinium | Tudorza® | R03BB05 |
|  | Glycopyrronium | Seebri® | R03BB06 |
|  | Tiotropium | Spiriva® | R03BB04 |
|  | Umeclidinium | Incruse® | R03BB07 |
| LTRA | Montelukast | Singulair® | R03DC03 |
|  | Zafirlukast | Accolate® | R03DC01 |
| SABA | Salbutamol | Ventolin® | R03CC02 |
|  | Terbutaline Sulfate | Bricanyl® | R03CC03 |
| SAMA | Ipratropium Bromide | Atrovent® | R03BB01 |
| ICS=Inhaled Corticosteroid, LABA= Long-acting β-agonist, LAMA = Long-acting muscarinic antagonist, LTRA=Leukotriene receptor antagonist, SABA=Short-acting β-agonist, SAMA= Short-acting muscarinic antagonist | | | |

**Table S2.** Additional Sample Characteristics (n=743)

| **Symptom** | **No**  **n (%)** | **Yes**  **n (%)** | **N/A**  **n (%)** |
| --- | --- | --- | --- |
| Breathlessness | 286 (39) | 350 (47) | 107 (14) |
| Chest Tightness | 61 (8) | 202 (27) | 477 (64) |
| Cough | 131 (18) | 500 (67) | 112 (15) |
| Sputum | 121 (16) | 342 (46) | 276 (37) |
| Wheeze | 400 (54) | 186 (25) | 150 (150) |
| Symptoms vary over time | 197 (27) | 333 (45) | 212 (28) |
| Symptoms worsen at night/morning | 365 (49) | 152 (21) | 224 (30) |
| Symptoms triggered by allergen/exercise/irritant | 247 (33) | 277 (37) | 218 (29) |
| Symptoms appear or worsen with viral infection | 63 (9) | 464 (62) | 215 (29) |
| Symptoms respond to therapy/medication | 10 (1) | 185 (25) | 543 (73) |
| **Asthma Exacerbations** | | **n (%)** | |
| Recorded emergency department visit ever (for asthma) | | 16 (2) | |
| Recorded hospitalization ever (for asthma) | | 3 (0) | |
| Recorded systemic steroid use ever (for asthma) | | 30 (4) | |

**Table S3.** Case Definition Results (Combined Suspected or Confirmed Asthma)

| **Case Definition** | **TP** | **FP** | **FN** | **TN** | **SN**  **(95% CI)** | **SP**  **(95% CI)** | **PPV**  **(95% CI)** | **NPV**  **(95% CI)** | **YI**  **(95% CI)** |
| --- | --- | --- | --- | --- | --- | --- | --- | --- | --- |
| 1* | 25 | 7 | 47 | 664 | 0.35  (0.24-0.47) | 0.99  (0.98-1.00) | 0.78  (0.62-0.89) | 0.93  (0.92-0.94) | 0.34  (0.22-0.47) |
| 2 | 54 | 19 | 18 | 652 | 0.75  (0.63-0.84) | 0.97  (0.96-0.98) | 0.74  (0.64-0.82) | 0.97  (0.96-0.98 | 0.72  (0.59-0.82) |
| 3* | 3 | 2 | 69 | 669 | 0.04  (0.01-0.12) | 1.00  (0.99-1.00) | 0.60  (0.20-0.90) | 0.91  (0.90-0.91) | 0.04  (0.00-0.12) |
| 4 | 58 | 25 | 14 | 646 | 0.81  (0.70-0.89) | 0.96  (0.95-0.98) | 0.70  (0.61-0.78) | 0.98  (0.97-0.99) | 0.77  (0.65-0.87) |
| 5 | 58 | 24 | 14 | 647 | 0.81  (0.70-0.89) | 0.96  (0.94-0.98) | 0.71  (0.62-0.79) | 0.98  (0.97-0.99) | 0.77  (0.64-0.87) |
| 6 | 58 | 25 | 14 | 646 | 0.81  (0.70-0.89) | 0.96  (0.95-0.98) | 0.70  (0.61-0.78) | 0.98  (0.97-0.99) | 0.77  (0.65-0.87) |
| 7 | 58 | 24 | 14 | 647 | 0.81  (0.70-0.89) | 0.96  (0.94-0.98) | 0.71  (0.62-0.79) | 0.98  (0.97-0.99) | 0.77  (0.64-0.87) |
| 8 | 58 | 25 | 14 | 646 | 0.81  (0.70-0.89) | 0.96  (0.95-0.98) | 0.70  (0.61-0.78) | 0.98  (0.97-0.99) | 0.77  (0.65-0.87) |
| 9 | 58 | 24 | 14 | 647 | 0.81  (0.70-0.89) | 0.96  (0.94-0.98) | 0.71  (0.62-0.79) | 0.98  (0.97-0.99) | 0.77  (0.64-0.87) |
| 10 | 58 | 25 | 14 | 646 | 0.81  (0.70-0.89) | 0.96  (0.95-0.98) | 0.70  (0.61-0.78) | 0.98  (0.97-0.99) | 0.77  (0.65-0.87) |
| 11 | 58 | 24 | 14 | 647 | 0.81  (0.70-0.89) | 0.96  (0.94-0.98) | 0.71  (0.62-0.79) | 0.98  (0.97-0.99) | 0.77  (0.64-0.87) |
| 12 | 56 | 19 | 16 | 652 | 0.78  (0.66-0.87) | 0.97  (0.96-0.98) | 0.75  (0.65-0.82) | 0.98  (0.96-0.98) | 0.75  (0.62-0.85) |
| 13† | 56 | 19 | 16 | 652 | 0.78  (0.66-0.87) | 0.97  (0.96-0.98) | 0.75  (0.65-0.82) | 0.98  (0.96-0.98) | 0.75  (0.62-0.85) |
| 14 | 61 | 46 | 11 | 625 | 0.85  (0.74-0.92) | 0.93  (0.91-0.95) | 0.57  (0.50-0.64) | 0.98  (0.97-0.99) | 0.78  (0.65-0.87) |
| M-1 | 25 | 28 | 47 | 643 | 0.35  (0.24-0.47) | 0.96  (0.94-0.97) | 0.47  (0.36-0.59) | 0.93  (0.92-0.94) | 0.31  (0.18-0.44) |
| M-2 | 7 | 11 | 65 | 660 | 0.10  (0.04-0.19) | 0.98  (0.97-0.99) | 0.39  (0.20-0.61) | 0.91  (0.90-0.92) | 0.08  (0.01-0.18) |
| M-3 | 6 | 13 | 66 | 658 | 0.08  (0.03-0.17) | 0.98  (0.97-0.99) | 0.32  (0.15-0.54) | 0.91  (0.90-0.91) | 0.06  (0.01-0.16) |
| M-4 | 13 | 1 | 59 | 670 | 0.18  (0.10-0.29) | 0.99  (0.99-1.00) | 0.93  (0.63-0.99) | 0.92  (0.91-0.93) | 0.17  (0.09-0.29) |
| M-5 | 6 | 3 | 66 | 668 | 0.08  (0.03-0.17) | 0.99  (0.99-1.00) | 0.67  (0.34-0.89) | 0.91  (0.90-0.92) | 0.07  (0.02-0.17) |
| M-6 | 0 | 0 | 90 | 653 | 0.00  (0.00-0.04) | 1.00  (0.99-1.00) | - | 0.90  (0.90-0.90) | 0.00  (0.00-0.04) |
| M-7 | 55 | 67 | 17 | 604 | 0.76  (0.65-0.86) | 0.90  (0.87-0.92) | 0.45  (0.39-0.52) | 0.97  (0.96-0.98) | 0.66  (0.52-0.78) |
| *Adapted from Xi et al. (2015)  †Adapted from Cave et al. (2020)  TP=True Positive, FP=False Positive, FN=False Negative, TN=True Negative, SN=Sensitivity, SP=Specificity, PPV=Positive Predictive Value, NPV=Negative Predictive Value, YI=Youden’s Index | | | | | | | | | |

**Table S4.** Case Definition Results (Suspected Asthma)

| **Case Definition** | **TP** | **FP** | **FN** | **TN** | **SN**  **(95% CI)** | **SP**  **(95% CI)** | **PPV**  **(95% CI)** | **NPV**  **(95% CI)** | **YI**  **(95% CI)** |
| --- | --- | --- | --- | --- | --- | --- | --- | --- | --- |
| 1* | 14 | 18 | 40 | 671 | 0.26  (0.15-0.40) | 0.97  (0.96-0.98) | 0.44  (0.29-0.60) | 0.94  (0.94-0.95) | 0.23  (0.11-0.38) |
| 2 | 38 | 35 | 16 | 654 | 0.70  (0.56-0.82) | 0.95  (0.93-0.96) | 0.52  (0.43-0.61) | 0.98  (0.96-0.98) | 0.65  (0.49-0.78) |
| 3* | 3 | 2 | 51 | 687 | 0.06  (0.01-0.15) | 1.00  (0.99-1.00) | 0.60  (0.20-0.90) | 0.93  (0.93-0.94) | 0.05  (0.00-0.15) |
| 4 | 41 | 42 | 13 | 647 | 0.76  (0.62-0.87) | 0.94  (0.92-0.96) | 0.49  (0.41-0.57) | 0.98  (0.97-0.99) | 0.70  (0.54-0.83) |
| 5 | 41 | 41 | 13 | 648 | 0.76  (0.62-0.87) | 0.94  (0.92-0.96) | 0.50  (0.42-0.58) | 0.98  (0.97-0.99) | 0.70  (0.54-0.83) |
| 6 | 41 | 42 | 13 | 647 | 0.76  (0.62-0.87) | 0.94  (0.92-0.96) | 0.49  (0.41-0.57) | 0.98  (0.97-0.99) | 0.70  (0.54-0.83) |
| 7 | 41 | 41 | 13 | 648 | 0.76  (0.62-0.87) | 0.94  (0.92-0.96) | 0.50  (0.42-0.58) | 0.98  (0.97-0.99) | 0.70  (0.54-0.83) |
| 8 | 41 | 42 | 13 | 647 | 0.76  (0.62-0.87) | 0.94  (0.92-0.96) | 0.49  (0.41-0.57) | 0.98  (0.97-0.99) | 0.70  (0.54-0.83) |
| 9 | 41 | 41 | 13 | 648 | 0.76  (0.62-0.87) | 0.94  (0.92-0.96) | 0.50  (0.42-0.58) | 0.98  (0.97-0.99) | 0.70  (0.54-0.83) |
| 10 | 41 | 42 | 13 | 647 | 0.76  (0.62-0.87) | 0.94  (0.92-0.96) | 0.49  (0.41-0.57) | 0.98  (0.97-0.99) | 0.70  (0.54-0.83) |
| 11 | 41 | 41 | 13 | 648 | 0.76  (0.62-0.87) | 0.94  (0.92-0.96) | 0.50  (0.42-0.58) | 0.98  (0.97-0.99) | 0.70  (0.54-0.83) |
| 12 | 40 | 35 | 14 | 654 | 0.74  (0.60-0.85) | 0.95  (0.93-0.96) | 0.53  (0.44-0.62) | 0.98  (0.97-0.99) | 0.69  (0.53-0.81) |
| 13† | 40 | 35 | 14 | 654 | 0.74  (0.60-0.85) | 0.95  (0.93-0.96) | 0.53  (0.44-0.62) | 0.98  (0.97-0.99) | 0.69  (0.53-0.81) |
| 14 | 44 | 63 | 10 | 626 | 0.81  (0.69-0.91) | 0.91  (0.88-0.93) | 0.41  (0.35-0.48) | 0.98  (0.97-0.99) | 0.72  (0.57-0.84) |
| M-1 | 2 | 6 | 52 | 683 | 0.09  (0.00-0.13) | 0.99  (0.98-1.00) | 0.25  (0.06-0.61) | 0.93  (0.93-0.93) | 0.08  (0.00-0.13) |
| M-2 | 0 | 1 | 54 | 671 | 0.00  (0.00-0.05) | 1.00  (0.99-1.00) | - | 0.93  (0.93-0.93) | 0.00  (0.00-0.05) |
| M-3 | 11 | 26 | 43 | 663 | 0.20  (0.11-0.34) | 0.96  (0.95-0.98) | 0.30  (0.18-0.44) | 0.94  (0.93-0.95) | 0.16  (0.02-0.23) |
| M-4 | 8 | 6 | 46 | 683 | 0.15  (0.07-0.27) | 0.99  (0.98-1.00) | 0.57  (0.32-0.79) | 0.94  (0.93-0.94) | 0.14  (0.05-0.27) |
| M-5 | 3 | 6 | 51 | 683 | 0.06  (0.01-0.15) | 0.99  (0.98-1.00) | 0.33  (0.11-0.66) | 0.93  (0.93-0.94) | 0.05  (0.00-0.15) |
| M-6 | 0 | 0 | 54 | 689 | 0.00  (0.00-0.07) | 1.00  (0.99-1.00) | - | 0.93  (0.93-0.93 | 0.00  (0.00-0.07) |
| M-7 | 20 | 51 | 34 | 638 | 0.37  (0.24-0.51) | 0.93  (0.90-0.94) | 0.28  (0.20-0.38) | 0.95  (0.94-0.96) | 0.30  (0.14-0.44) |
| *Adapted from Xi et al. (2015)  †Adapted from Cave et al. (2020)  TP=True Positive, FP=False Positive, FN=False Negative, TN=True Negative, SN=Sensitivity, SP=Specificity, PPV=Positive Predictive Value, NPV=Negative Predictive Value, YI=Youden’s Index | | | | | | | | | |

**Table S5.** Case Definition Results (Confirmed Asthma)

| **Case Definition** | **TP** | **FP** | **FN** | **TN** | **SN**  **(95% CI)** | **SP**  **(95% CI)** | **PPV**  **(95% CI)** | **NPV**  **(95% CI)** | **YI**  **(95% CI)** |
| --- | --- | --- | --- | --- | --- | --- | --- | --- | --- |
| 1* | 11 | 21 | 7 | 704 | 0.61  (0.38-0.83) | 0.97  (0.96-0.98) | 0.34  (0.23-0.48) | 0.99  (0.98-0.99) | 0.58  (0.34-0.81) |
| 2 | 16 | 57 | 2 | 668 | 0.89  (0.65-0.98) | 0.92  (0.90-0.94) | 0.22  (0.17-0.27) | 1.00  (0.99-1.00) | 0.81  (0.55-0.92) |
| 3* | 0 | 5 | 18 | 720 | 0.00  (0.00-0.19) | 0.99  (0.98-1.00) | - | 0.98  (0.98-.0.98) | 0.00  (0.00-0.19) |
| 4 | 17 | 66 | 1 | 659 | 0.94  (0.73-1.00) | 0.91  (0.89-0.93) | 0.20  (0.17-0.25) | 1.00  (0.99-1.00) | 0.85  (0.62-0.93) |
| 5 | 17 | 65 | 1 | 660 | 0.94  (0.73-1.00) | 0.91  (0.89-0.93) | 0.21  (0.17-0.25) | 1.00  (0.99-1.00) | 0.85  (0.62-0.93) |
| 6 | 17 | 66 | 1 | 659 | 0.94  (0.73-1.00) | 0.91  (0.89-0.93) | 0.20  (0.17-0.25) | 1.00  (0.99-1.00) | 0.85  (0.62-0.93) |
| 7 | 17 | 65 | 1 | 660 | 0.94  (0.73-1.00) | 0.91  (0.89-0.93) | 0.21  (0.17-0.25) | 1.00  (0.99-1.00) | 0.85  (0.62-0.93) |
| 8 | 17 | 66 | 1 | 659 | 0.94  (0.73-1.00) | 0.91  (0.89-0.93) | 0.20  (0.17-0.25) | 1.00  (0.99-1.00) | 0.85  (0.62-0.93) |
| 9 | 17 | 65 | 1 | 660 | 0.94  (0.73-1.00) | 0.91  (0.89-0.93) | 0.21  (0.17-0.25) | 1.00  (0.99-1.00) | 0.85  (0.62-0.93) |
| 10 | 17 | 66 | 1 | 659 | 0.94  (0.73-1.00) | 0.91  (0.89-0.93) | 0.20  (0.17-0.25) | 1.00  (0.99-1.00) | 0.85  (0.62-0.93) |
| 11 | 17 | 65 | 1 | 660 | 0.94  (0.73-1.00) | 0.91  (0.89-0.93) | 0.21  (0.17-0.25) | 1.00  (0.99-1.00) | 0.85  (0.62-0.93) |
| 12 | 16 | 59 | 2 | 666 | 0.89  (0.65-0.99) | 0.92  (0.90-0.94) | 0.21  (0.17-0.27) | 1.00  (0.99-1.00) | 0.81  (0.55-0.93) |
| 13† | 16 | 59 | 2 | 666 | 0.89  (0.65-0.99) | 0.92  (0.90-0.94) | 0.21  (0.17-0.27) | 1.00  (0.99-1.00) | 0.81  (0.55-0.93) |
| 14 | 17 | 90 | 1 | 635 | 0.94  (0.73-1.00) | 0.88  (0.85-0.90) | 0.16  (0.13-0.19) | 1.00  (0.99-1.00) | 0.82  (0.58-0.90) |
| M-1 | 5 | 48 | 13 | 677 | 0.28  (0.10-0.53) | 0.93  (0.91-0.95) | 0.09  (0.05-0.19) | 0.98  (0.98-0.99) | 0.21  (0.01-0.48) |
| M-2 | 1 | 17 | 17 | 708 | 0.06  (0.00-0.27) | 0.98  (0.96-0.99) | 0.06  (0.01-0.30) | 0.98  (0.97-0.98) | 0.04  (0.00-0.26) |
| M-3 | 2 | 17 | 16 | 708 | 0.11  (0.01-0.35) | 0.98  (0.96-0.99) | 0.11  (0.03-0.32) | 0.98  (0.97-0.98) | 0.09  (0.00-0.34) |
| M-4 | 5 | 9 | 13 | 716 | 0.28  (0.10-0.53) | 0.99  (0.98-1.00) | 0.36  (0.17-0.60) | 0.98  (0.98-0.99) | 0.27  (0.08-0.53) |
| M-5 | 3 | 6 | 15 | 719 | 0.17  (0.04-0.41) | 0.99  (0.98-1.00) | 0.33  (0.12-0.65) | 0.98  (0.98-0.98) | 0.16  (0.02-0.41) |
| M-6 | 0 | 0 | 18 | 725 | 0.00  (0.00-0.19) | 1.00  (0.99-1.00 | - | 0.98  (0.98-0.98) | 0.00  (0.00-0.19) |
| M-7 | 17 | 105 | 1 | 620 | 0.94  (0.73-1.00) | 0.86  (0.83-0.88) | 0.14  (0.12-0.17) | 1.00  (0.99-1.00 | 0.80  (0.56-0.88) |
| *Adapted from Xi et al. (2015)  †Adapted from Cave et al. (2020)  TP=True Positive, FP=False Positive, FN=False Negative, TN=True Negative, SN=Sensitivity, SP=Specificity, PPV=Positive Predictive Value, NPV=Negative Predictive Value, YI=Youden’s Index | | | | | | | | | |
